# Association between high expression of intratumoral fibroblast activation protein and survival in patients with intrahepatic cholangiocarcinoma

**DOI:** 10.1186/s12876-023-03012-x

**Published:** 2023-11-28

**Authors:** Yuhei Waki, Yuji Morine, Takayuki Noma, Chie Takasu, Hiroki Teraoku, Shinichiro Yamada, Yu Saito, Tetsuya Ikemoto, Mitsuo Shimada

**Affiliations:** Department of Digestive and Transplant Surgery, Tokushima University, 3-18-15 Kuramoto-cho, Tokushima, 770-8503 Japan

**Keywords:** Fibroblast activation protein, Cancer-associated fibroblast, Intrahepatic cholangiocarcinoma, Prognostic factor

## Abstract

**Background:**

Cancer-associated fibroblasts (CAFs) have been reported to exhibit protumorigenic effects. Among the well-known CAF markers such as smooth muscle actin (SMA) and fibroblast activation protein (FAP), high expression of SMA in the peritumoral stroma has been reported to be a prognostic factor in various cancers. However, the effect of high FAP expression in intrahepatic cholangiocarcinoma (IHCC) has not been fully clarified. We evaluated the expression of CAF markers, focusing on FAP expression in the peripheral and intratumoral regions, to clarify the association with survival in patients with IHCC.

**Methods:**

The study cohort comprised 37 patients who underwent curative resection for IHCC. The FAP expressions were evaluated in the peripheral and intratumoral regions of the resected tissues. Clinicopathological factors and survival outcomes were investigated between patients with high versus low FAP expression. Uni- and multivariate analyses were performed to identify the prognostic factors for overall survival and relapse-free survival.

**Results:**

The median area percentages of FAP expression in the peripheral and intratumoral regions were 15.5% and 17.8%, respectively. High FAP expression in the intratumoral region was significantly associated with worse overall survival and disease-free survival than low FAP expression in the intratumoral region. Multivariate analysis identified high intratumoral FAP expression as a risk factor for worse overall survival (hazard ratio, 2.450; *p* = 0.049) and relapse-free survival (hazard ratio, 2.743; *p* = 0.034).

**Conclusions:**

High intratumoral FAP expression was associated with worse survival, suggesting that intratumoral FAP expression represents malignant progression in patients with IHCC.

**Supplementary Information:**

The online version contains supplementary material available at 10.1186/s12876-023-03012-x.

## Introduction

Intrahepatic cholangiocarcinoma (IHCC) is the second most common primary liver malignant tumor, and its incidence and mortality are increasing worldwide [1]. Surgical resection is the most effective treatment for resectable IHCC; however, even after curative resection, IHCC has a high recurrence rate and poor prognosis [2]. Recently, molecular targeting therapies and immune checkpoint inhibitors are expected to become treatment options for IHCC. Therefore, the exploration of molecular targets with clinical and molecular classifications may aid in the establishment of a treatment strategy for IHCC.

The tumor microenvironment (TME) has drawn increasing attention in predicting cancer prognosis and metastasis in several types of cancer, such as prostate cancer [3], pancreatic ductal adenocarcinoma [4], esophageal squamous cell carcinoma [5], breast cancer [6], and oral squamous cell carcinoma [7]. The TME is composed of cancer-associated fibroblasts (CAFs), tumor-activated macrophages, immune cells, an extracellular matrix, and cancer cells, which interact with each other and release growth factors, extracellular matrix proteins, and angiogenic factors [8–10]. Malignant enhancement induced by the TME has been demonstrated in in vitro models of a variety of carcinomas [6, 8].

CAFs have been reported to play an important role in the TME and may determine the cancer cell behavior [11]. Generally, CAFs are known to exhibit protumorigenic effects by promoting cancer cell proliferation and invasion. Furthermore, the CAF maturation qualified by the expressions of fibroblast activation protein (FAP) and α-smooth muscle actin (SMA) has a significant impact on tumor growth and progression [12, 13]. However, these markers do not mark all CAFs, and FAP is not only selectively expressed by CAFs in most human epithelial cancers, but also by reactive stromal fibroblasts under certain inflammatory conditions, such as liver cirrhosis. [14, 15]. A recent study also revealed the pivotal role of FAP-positive CAF in shaping the optimal supportive niche for IHCC by mediating hyperactivated 5-lipoxygenase (5-LO) of myeloid-derived suppressor cells (MDSCs) in the TME [16]. Further study showed that FAP-positive CAFs promoted proinflammatory gene expression and may be involved in tumor immune evasion in a mouse model of pancreatic cancer [17]. Despite the fact that FAP is selectively expressed in CAFs and often used as a CAF marker, it remains largely unclear whether the histopathologic expression patterns and intensity of FAP play a role in clinical outcomes in IHCC. Therefore, we aimed to evaluate the intensity and patterns of FAP expression to clarify the association with survival in patients with IHCC. Moreover, the correlation between FAP-positive CAFs and the expression of 5-LO, which is thought to be involved in tumor immunity, was examined.

## Materials and methods

### Patients

Of the 61 patients who underwent initial hepatectomy for IHCC at Tokushima University Hospital between April 1997 and July 2020, we excluded eight patients who received non-curative surgery, one patient who received preoperative chemotherapy, and 15 patients whose surgical specimens were unavailable. Accordingly, a total of 37 patients were enrolled in this study.

Clinicopathological parameters were obtained from the medical records. Staging was defined in accordance with the Classification of Primary Liver Cancer by the Liver Cancer Study Group of Japan. Curability was defined as follows: curability A, no residual tumor for stage I and II patients; curability B, no residual tumor for stage III and IV patients; and curability C, definite residual tumors. This study was approved by the institutional review board of Tokushima University Hospital (approval no. 3215).

### Analyzed factors

The following clinicopathological variables were collected: age, sex, history of hepatitis B or C infection, tumor markers, pathological TNM classification, and compliance with adjuvant chemotherapy. The assessed surgical outcomes were the type of hepatectomy, extent of lymph node dissection, operation time, and blood loss. The postoperative outcomes were categorized in accordance with the Clavien-Dindo classification (CD). Postoperative complications were defined as any adverse events corresponding to CD grade II or above that occurred during postoperative hospitalization. Overall survival (OS) and recurrence-free survival (RFS) were also evaluated.

### Immunohistochemistry procedures

FAP (dilution 1:250, ab207178; Abcam PLC, Cambridge, UK), SMA (dilution 1:100, ab7818; Abcam PLC), and 5-LO (dilution 1:300, ab169755; Abcam PLC) were used as the primary antibodies. The immunohistochemistry procedures were performed as previously reported [18]. Briefly, specimens were fixed in 10% formalin, embedded in paraffin, and sliced into 5 μm-thick serial sections. Slides were then dewaxed, deparaffinized with xylene, and rehydrated with a stepwise reduction in alcohol concentration. Next, the slides were boiled with citrate or ethylenediaminetetraacetic acid buffer for 20 min in a microwave oven to activate the antigen. To prevent nonspecific antigen binding, endogenous peroxidases were blocked with 0.3% hydrogen peroxide for 30 min, followed by incubation in 5% goat serum for 1 h. The slides were then incubated with primary indicated antibodies overnight at 4 °C. A secondary peroxidase-labeled polymer conjugated with goat anti-mouse immunoglobulin was coated for 1 h. The sections were developed with 3,3-diaminobenzidine (DAB) and counterstained with Mayer’s hematoxylin. Finally, each slide was dehydrated using a graded series of alcohol concentrations and covered with a coverslip.

### FAP and SMA area calculations and 5-LO cell counts

The areas of FAP and SMA expression were identified by screening the entire tumor area in a low-powered field (40× magnification) and were randomly evaluated in three areas (200× magnification) in the peripheral region (within one high-powered field inside and outside of the tumor margin) and intratumoral region (tumor center more than one high-powered field away from the tumor margin). Based on a previous study [11], we analyzed the images using Image J (software ver. 1.53, National Institutes of Health, Bethesda, MD, USA) and the color deconvolution plugin (http://imagej.net/Colour_Deconvolution) for ImageJ and Fiji to implement staining separation via the method of Ruifrok and Johnston [19]. The FAP- and SMA-positive areas were detected as a brown color under DAB staining. The brown positive area was extracted with the color deconvolution plugin (vector: H DAB), and the area was measured using the adjusted thresholds for SMA (upper cutoff, 210; lower cutoff, 0) and for FAP (upper cutoff, 220; lower cutoff, 0). The area percentages of FAP and SMA were calculated as the ratio of the positive-stained areas relative to the total areas.

Round cells positive for 5-LO were identified by screening the entire tumor area in a low-powered field (100× magnification) and were randomly evaluated in five high-powered field areas (400× magnification) in the peripheral and intratumoral regions. The mean number of 5-LO-positive cells was calculated. The pathological evaluation was performed by two investigators who were blinded to the patients’ backgrounds and prognoses.

### Statistical analysis

All statistical analyses were performed using R version 4.2.0 software package. Continuous data are presented as median (interquartile range [IQR]). Continuous variables were non-parametrically analyzed using the Mann-Whitney test. Categorical variables were compared using the Chi-squared test or Fisher exact test. OS and RFS were analyzed using the Kaplan-Meier estimation, and differences in survivals were compared using the log-rank test. Variables with a *p* value of less than 0.05 in the univariate analyses were included in the multivariate Cox regression analysis. *P* values of less than 0.05 were considered statistically significant.

**Fig. 1:**
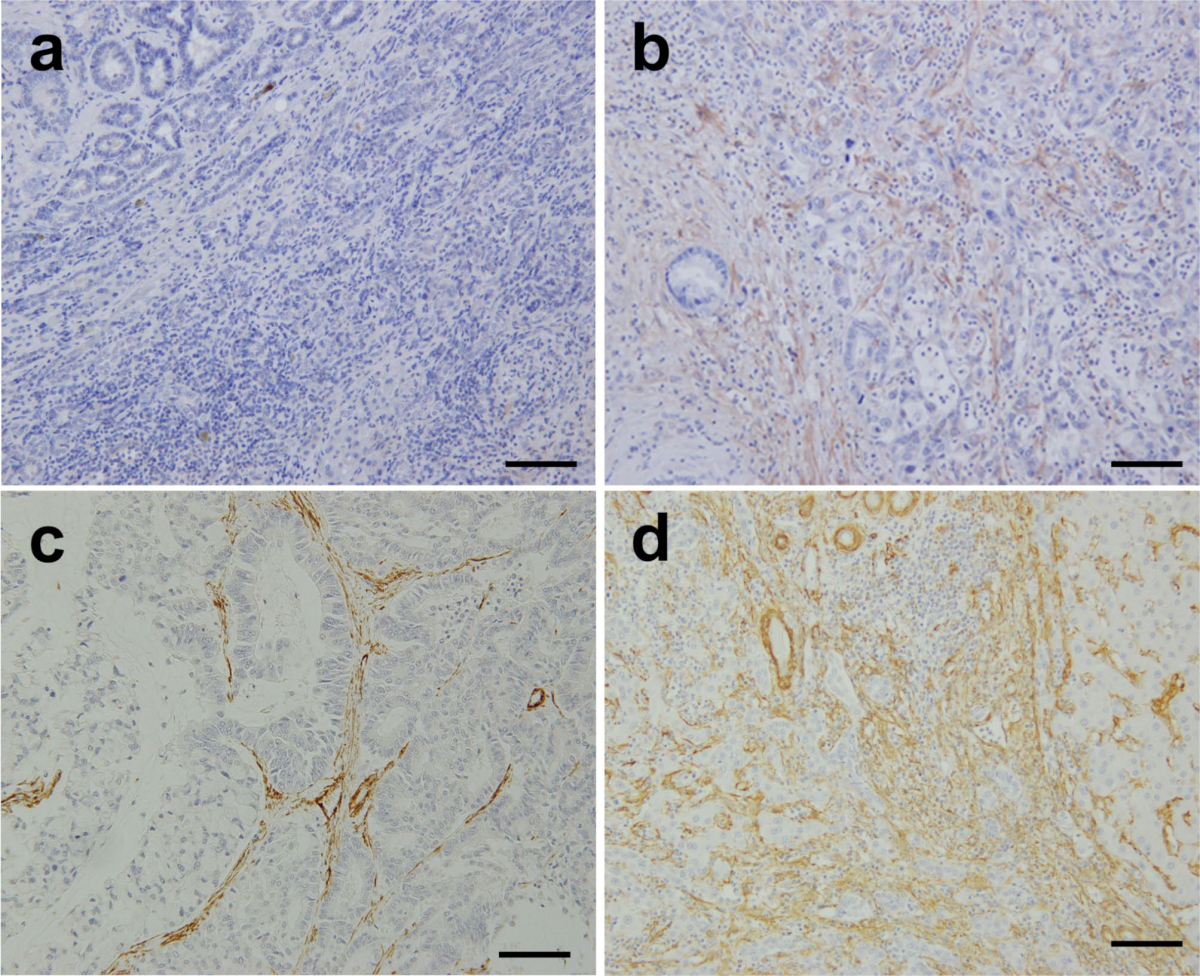
Immunohistochemical staining of IHCC tissues for FAP and SMA. Staining showing (a) low and (b) high expressions of FAP (magnification ×200). Staining showing (c) low and (d) high expressions of SMA (magnification ×200). Bar = 100 μm. IHCC, intrahepatic cholangiocarcinoma; FAP, fibroblast activation protein; SMA, smooth muscle actin

## Results

### Immunohistochemistry of FAP and SMA

Representative images of low and high expressions of FAP and SMA are shown in Fig. 1. The median area percentages of FAP and SMA in the peripheral regions were 15.5% (IQR, 11.7–22.4) and 13.6% (IQR, 12.0–17.3), respectively. The median area percentages of FAP and SMA in the intratumoral regions were 17.8% (IQR, 11.8–24.0) and 9.57% (IQR, 7.23–13.1), respectively.

**Table 1:** Clinicopathological findings and surgical outcomes of the low and high intratumoral FAP groups

Factors	Low FAP (n = 10)	High FAP (n = 27)	P value
Age [years; median (IQR)]	70 (64.3–75.5)	71 (63.5–76.0)	0.837
Gender, n (%)			0.275
Female	5 (50.0)	8 (29.6)	
Male	5 (50.0)	19 (70.4)	
History of hepatitis B infection, n (%)	2 (20.0)	8 (29.6)	0.694
History of hepatitis C infection, n (%)	1 (10.0)	4 (14.8)	1.000
CEA [mg/dL; median (IQR)]	2.70 (1.83–8.63)	2.60 (1.50–5.75)	0.656
CA19-9 [mg/dL; median (IQR)]	28.5 (10.0–95.0)	187.0 (15.5–1016.0)	0.171
Type of hepatectomy, n (%)			0.921
Hr0	1 (10.0)	1 (3.7)	
Hr1	2 (20.0)	6 (22.2)	
Hr2	6 (60.0)	17v(63.0)	
Hr3	1 (10.0)	3 (11.1)	
Lymph nodes dissection			0.881
D0	6 (60.0)	13 (48.1)	
D2 or D2+	4 (40.0)	14 (51.9)	
Operation time [min; median (IQR)]	324.0 (275.5-353.8)	344.0 (287.0-438.0)	0.365
Blood loss [mL; median (IQR)]	122.5 (111.0-204.2)	285 (159.5-576.5)	0.067
All complications, yes, n (%)	1 (10.0)	6 (22.2)	0.647
Pathological tumor size [mm; median (IQR)]	54.0 (41.8–60.0)	35.0 (28.5–51.5)	0.057
Pathological T stage ^a^, n (%)			0.098
pT1	0 (0)	1 (3.7)	
pT2	7 (70.0)	7 (25.9)	
pT3	3 (30.0)	15 (55.5)	
pT4	0 (0.0)	4 (14.8)	
Pathological N stage ^a^, n (%)			0.393
pN0	9 (90.0)	19 (70.3)	
pN1	1 (10.0)	1 (3.7)	
Pathological stage ^a^, n (%)			0.036*
I	0 (0.0)	1 (3.7)	
II	7 (70.0)	5 (18.5)	
III	2 (20.0)	11 (40.7)	
IV	1 (10.0)	10 (37.0)	
Curability ^a^, n (%)			0.023*
A	7 (70.0)	7 (25.9)	
B	3 (30.0)	20 (74.1)	
Adjuvant chemotherapy, yes, n (%)	1 (10.0)	6 (22.2)	0.647
Peripheral region			
FAP (%)	38.4 (30.6–22.4)	15.5 (11.7–22.4)	0.296
SMA (%)	12.7 (11.5–14.1)	14.5 (12.7–19.2)	0.105
Intratumoral region			
SMA (%)	8.28 (5.94–13.8)	9.95 (8.23-13.0)	0.408

IQR, interquartile range; FAP, fibroblast activation protein; SMA, α-smooth muscle actin; CEA, carcinoembryonic antigen

^a^Classification of Primary Liver Cancer by the Liver Cancer Study Group of Japan

*Statistically significant

Supplemental Fig. 1 shows the receiver operating characteristic (ROC) curve used to identify the optimal cut-off value of intratumoral FAP expression to predict OS. The area under the curve (AUC) was 0.706 and the threshold of intratumoral FAP expression was 11.8% using the maximum value of (sensitivity + specificity – 1) [20], equivalent to a 25% tile of intratumor FAP expression. Hence, the 25th percentile values of FAP and SMA in both the peripheral and intratumoral regions were selected as the optimal cutoff values.

### Study population

Among the 37 patients, 10 and 27 were classified into the low and high intratumoral FAP expression groups, respectively. The clinicopathological findings and surgical outcomes of patients with low and high intratumoral FAP expression are summarized in Table 1. Regarding the clinicopathological findings, the patients with high intratumoral FAP expression had a worse pathological stage and curability than those with low intratumoral FAP expression. There were no significant differences in surgical outcomes between the two groups.

**Fig. 2:**
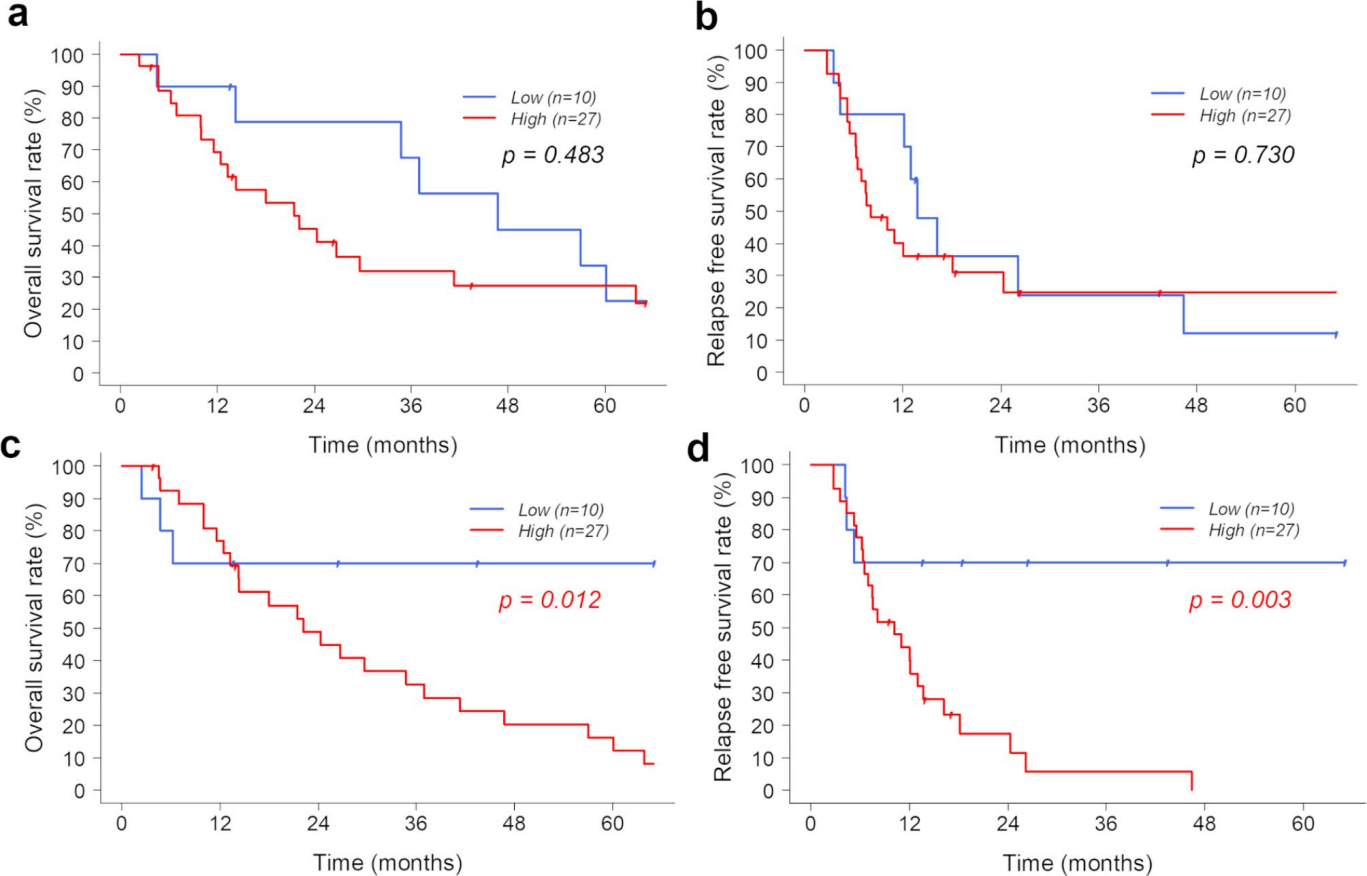
OS and RFS of the groups with low and high expressions of FAP in the peripheral and intratumoral regions. (a, b) There are no significant differences in OS and RFS between the groups with high and low FAP expressions in the peripheral region. (c, d) OS and RFS are significantly worse in the group with high FAP expression in the intratumoral region than the group with low FAP expression in the intratumoral region (*p* = 0.012 and *p* = 0.003, respectively). OS, overall survival; RFS, recurrence-free survival; FAP, fibroblast activation protein

### Survival analyses

The median follow-up period for all enrolled patients was 22.1 months (range, 2.3–147.7; IQR, 11.5–46.7). The OS and RFS were not significantly different between the patients with high FAP and low FAP expression in the peripheral region (*p* = 0.483 and p = 0.730, respectively), whereas the OS and RFS were significantly worse in the patients with high FAP expression in the intratumoral region than those with low FAP expression in the intratumoral region (*p* = 0.012 and p = 0.003, respectively) (Fig. 2a and d).

**Fig. 3:**
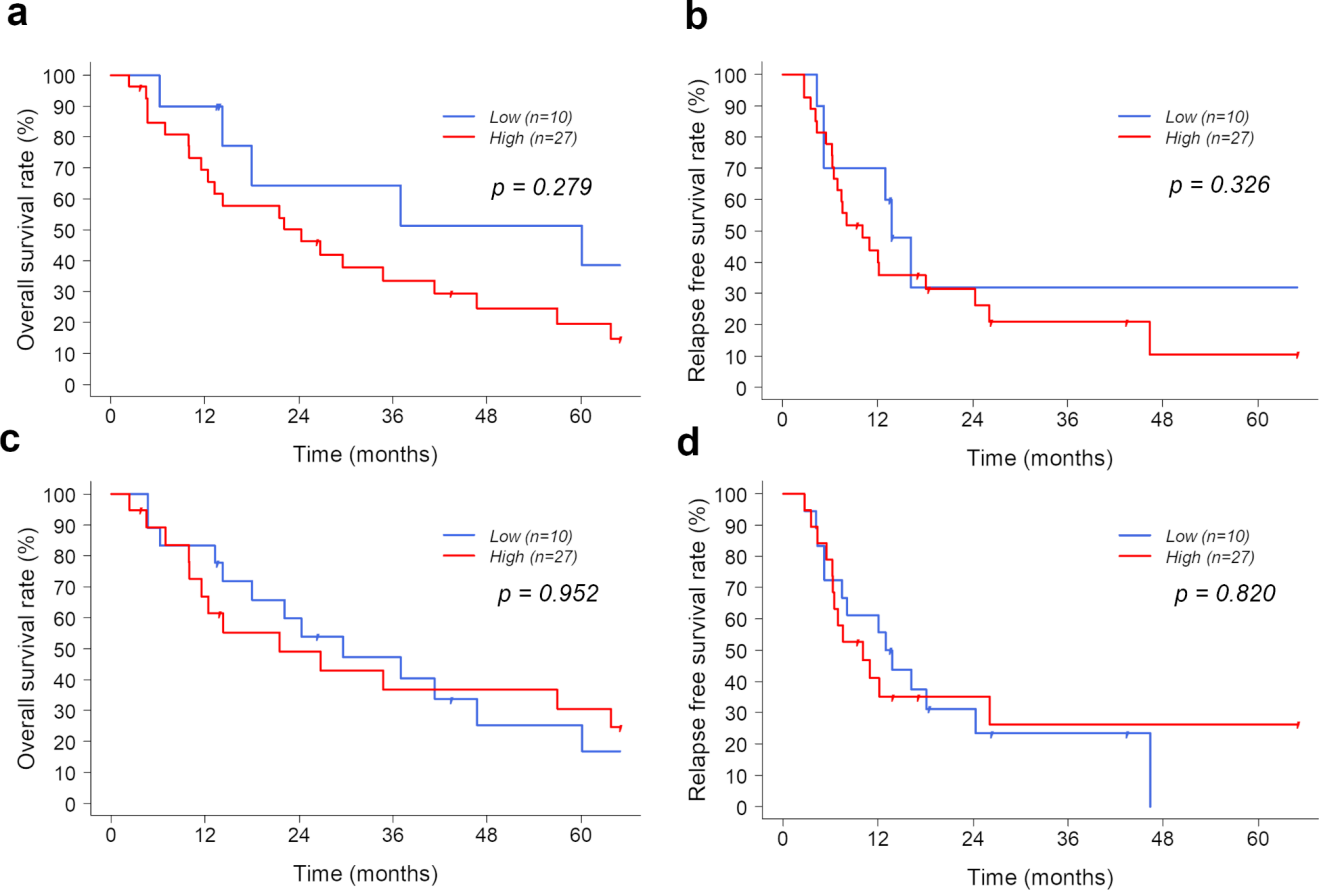
OS and RFS of the groups with low and high expressions of SMA in the peripheral and intratumoral regions. (a, b) OS tend to be lower in the group with high SMA expression than the group with low SMA expression in the peripheral region, but there was no significance in the RFS (p = 0.279 and p = 0.326, respectively). (c, d) There are no significant differences in OS and RFS between the groups with high and low expression of SMA in the intratumoral region. OS, overall survival; RFS, recurrence-free survival; SMA, smooth muscle actin

The OS tended to be worse in the patients with high SMA expression in the peripheral region than in those with low SMA expression in the peripheral region, but there was no significance in the RFS (*p* = 0.279 and *p* = 0.326, respectively) (Fig. 3a and b). There were no significant differences in the OS and RFS between the groups with high and low expressions of SMA in the intratumoral region (Fig. 3c and d).

### Uni- and multivariate survival analyses

The results of the uni- and multivariate analyses are shown in Table 2. Univariate analysis showed that pathological T category 4, positive lymph node metastasis, lack of adjuvant chemotherapy, and high FAP expression in the intratumoral region were associated with poor OS, while positive lymph node metastasis, lack of adjuvant chemotherapy, and high FAP expression in the intratumoral region were associated with poor RFS. Multivariate analysis showed that high FAP expression in the intratumoral region was an independent predictive factor of poor OS (hazard ratio, 2.450; *p* = 0.049) and RFS (hazard ratio, 2.743; *p* = 0.034) in patients with IHCC.

**Table 2:** Uni- and multivariate analyses of clinicopathological factors for overall and relapse-free survival

	Univariate			Multivariate		
	HR	95%CI	p	HR	95%CI	p
**Overall survival**						
Age, ≥ 70 vs. < 70	1.032	0.489–2.179	0.932			
Gender, male vs. female	0.584	0.237–1.434	0.170			
CEA, ≥ 5 vs. < 5	0.845	0.382–1.868	0.687			
CA19-9, ≥ 37 vs. < 37	1.952	0.930–4.097	0.075			
Complications, yes vs. no	1.619	0.564–4.646	0.280			
Adjuvant chemotherapy, no vs. yes	2.897	0.566–14.84	0.035*	2.029	0.698–5.893	0.194
Pathological T stage ^a^, 3/4 vs. 1/2	2.469	1.174–5.191	0.017*	1.845	0.723–4.706	0.200
Pathological N stage ^a^, 1 vs. 0	2.211	0.944–5.183	0.031*	1.243	0.466–3.318	0.664
Intratumoral FAP, high vs. low	3.303	1.523–7.162	0.013*	2.450	1.005–5.972	0.049*
**Recurrence-free survival**						
Age, ≥ 70 vs. < 70	0.939	0.446–1.977	0.862			
Gender, male vs. female	0.837	0.368–1.903	0.654			
CEA, ≥ 5 vs. < 5	0.792	0.362–1.737	0.571			
CA19-9, ≥ 37 vs. < 37	1.789	0.852–3.752	0.126			
Complications, yes vs. no	1.095	0.433–2.768	0.842			
Adjuvant chemotherapy, no vs. yes	3.139	1.228–8.026	0.001*	1.693	0.589–4.868	0.328
Pathological T stage ^a^, 3/4 vs. 1/2	2.564	1.218–5.396	0.009*	1.208	0.390–3.831	0.730
Pathological N stage ^a^, 1 vs. 0	2.664	0.947–7.499	0.009*	1.846	0.692–4.925	0.221
Intratumoral FAP, high vs. low	3.870	1.812–8.263	0.003*	2.591	1.034–6.493	0.042*

FAP, fibroblast activation protein; HR, hazard ratio; CI, confidence interval

^a^Classification of Primary Liver Cancer by the Liver Cancer Study

*Statistically significant

**Fig. 4:**
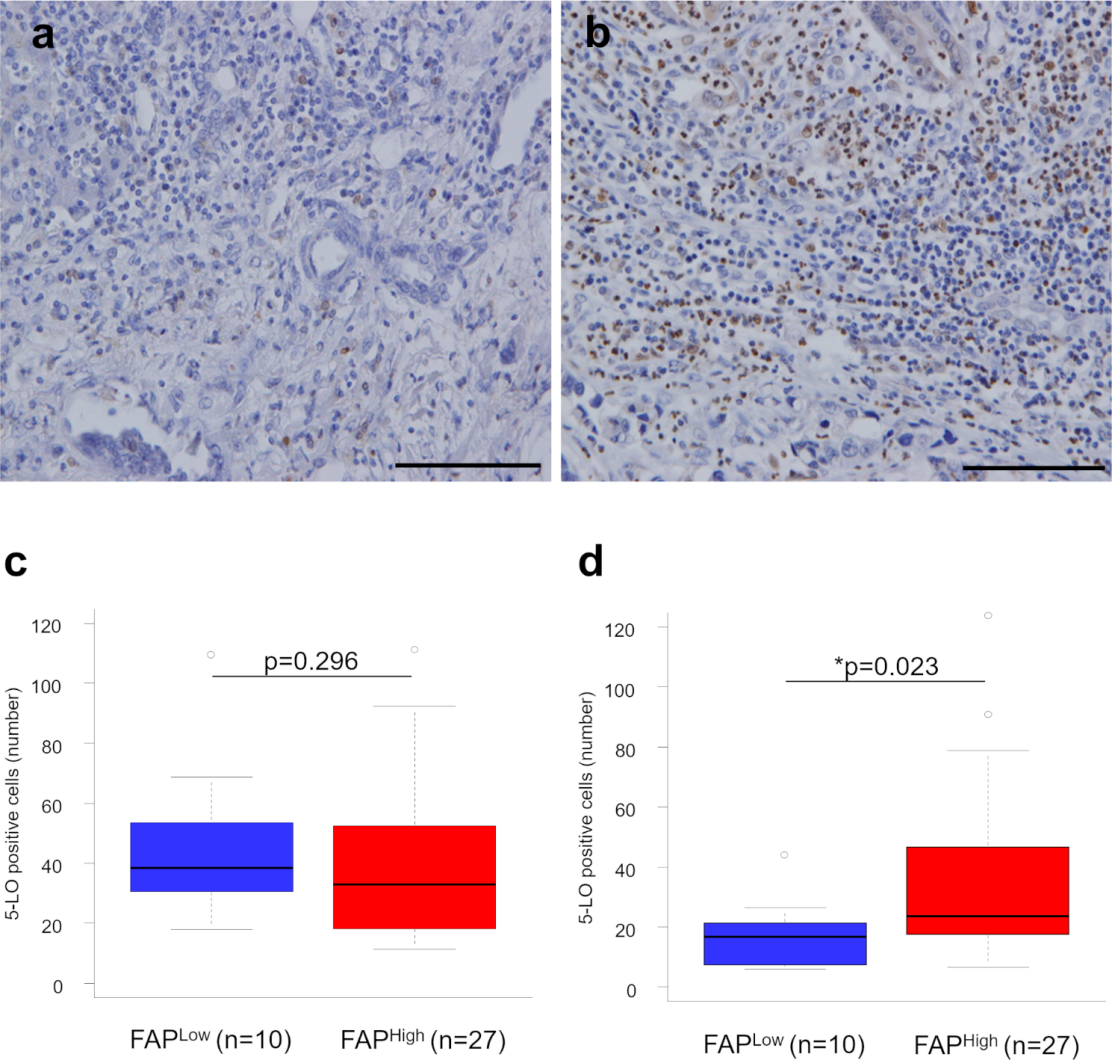
Immunohistochemical staining of IHCC tissues for 5-LO, and the association between the number of 5-LO-positive cells and FAP expression in the peripheral and intratumoral regions. Staining shows (a) low and (b) high expression of 5-LO (magnification ×400). Bar = 100 μm. (c) The number of 5-LO-positive cells is not significantly different between the groups with high and low FAP expression in the peripheral region. (d) The number of 5-LO-positive cells is significantly higher in the group with high FAP expression in the intratumoral region than the group with low FAP expression in the intratumoral region (*p* = 0.023). IHCC, intrahepatic cholangiocarcinoma; 5-LO, hyperactivated 5-lipoxygenase; FAP, fibroblast activation protein

### Associations between FAP and 5-LO in the peripheral and intratumoral regions

Representative images of low and high expressions of 5-LO are shown in Fig. 4a and b. The median number of 5-LO-positive cells in the peripheral region was 33.8 (IQR, 20.4–52.8). The median number of 5-LO-positive cells in the intratumoral region was 22.0 (IQR, 13.2–44.0).

The associations between the number of 5-LO-positive cells and the expression levels of FAP in the peripheral and intratumoral regions are shown in Fig. 4c and d. The number of 5-LO-positive cells was significantly higher in the group with high FAP expression in the intratumoral region than in the group with low FAP expression in the intratumoral region (*p* = 0.023).

## Discussion

The present study revealed that patients with IHCC with high intratumoral FAP expression had worse OS and RFS than those with low intratumoral FAP expression. In addition, high intratumoral FAP expression was identified as an independent prognostic factor for worse OS and RFS. In addition, the number of 5-LO-positive cells was significantly higher in the group with high FAP expression group in the intratumoral region than in the group with low FAP expression in the intratumoral region. In this study, we demonstrated that intratumoral high FAP expression in IHCC specimens has a negative effect on survival for the first time.

IHCC is a highly malignant cancer with poor survival due to a lack of evident symptoms in the early stage [21]. The paucity of appropriate medical approaches to IHCC other than surgical treatment has prompted further research into its clinical and biological characteristics [21]. A typical histological feature of IHCC is the presence of abundant fibrotic stroma that surrounds and infiltrates the tumor structures and a rich TME [22]. Several other cell types are recruited by IHCC cells and populate the TME, such as CAFs, endothelial and lymphatic cells, tumor-associated macrophages, and inflammatory cells. These cells contribute to IHCC progression and metastases through a variety of molecular mechanisms related to proliferation, migration, immunosuppression, and angiogenesis [21, 23]. Thus, exploration of clinical molecular targets in the TME may provide additional therapeutic strategies and improve the prognosis for IHCC.

CAFs interact with other cells, including endothelial cells and inflammatory cells, and secrete a variety of soluble factors including transforming growth factor-ß [24], platelet-derived growth factor receptor [25], hepatocyte growth factor [26], and fibroblast growth factor [27]. Recently, heterogeneity of CAFs has been reported in pancreatic cancers, with different functions such as myofibroblastic CAFs promoting tumor malignancy, inflammatory CAFs inducing inflammatory cytokines and immune cells, and antigen-presenting CAFs inducing regulatory T cells [28, 29]. Furthermore, CAFs activate numerous intracellular and paracrine signaling pathways to promote tumor growth and invasiveness [12]. Therefore, CAFs are one of the most prominent stromal components and play pivotal roles in modulating the TME.FAP has become known as an essential factor in CAFs, and high-level expression of FAP is associated with cancer proliferation and poor prognosis in various cancers, including IHCC, ovarian cancer and gastric cancer [30–32]. Notably, we demonstrated that high expression of FAP in the intratumoral region was associated with poor survival and was an independent prognostic factor for worse OS and RFS in curatively resected IHCC. FAP promotes the infiltration of MDSCs and tumor-infiltrating macrophages, which may create an immunosuppressive TME that antagonizes anti-tumor immunity [30, 33]. Previous studies demonstrated in mouse models of pancreatic cancer that FAP+/SMA- CAFs affected inflammation and that their enhancement leads to increased CD11b positive myeloid cells and worse prognosis, while FAP-/SMA + CAFs were associated with the decrease of Treg cells, potentially contributing to suppressing tumor progression [34]. Furthermore, the tumor-promoting effect of FAP-positive CAFs is partly mediated by attracting more MDSCs or macrophages to tumor sites, where they enhance the stemness of cancer cells and/or promote the expansion of cancer stem cells [17]. In addition, upregulation of FAP under hypoxia has been shown to cause epithelial-mesenchymal transition, which enhance stemness, invasiveness and metastasis of cancers, and poor prognosis in hepatocellular carcinoma [35]. Since IHCC generally has more fibrous components and less blood flow in the tumor compared to peripheral regions [22, 23], FAP-positive CAFs in the tumor center may promote hypoxia-induced epithelial-mesenchymal transition and stemness of cancer cells compared to the peripheral regions, leading to earlier tumor metastasis and recurrence. Therefore, our findings may provide new insights into the importance of FAP-positive cells in the intratumoral rather than the peripheral regions in evaluating the pathological tissue of IHCC. In addition, high FAP expression may constitute an immunosuppressive TME and represent worse survival.

SMA is the most well-accepted CAF marker, and high expression of SMA has been reported to be associated with poor survival in several gastrointestinal cancers, including hepatocellular carcinoma [36], pancreatic cancer [34], and cholangiocarcinoma [37]. Additionally, a previous study reported that enhanced interaction between the tumor and CAFs (as indicated by the enrichment of activated myofibroblasts with high SMA expression in peritumoral regions) promotes malignant behaviors [38]. Similarly, we demonstrated that high SMA expression in the peripheral region tended to be associated with poor survival compared with high SMA expression in the intratumoral region, although this association was not significant. Several studies have reported that high expression of SMA in the peripheral tumoral stroma is associated with the prognosis of patients with IHCC [15, 33]. Hence, high SMA expression in peripheral regions may be useful in identifying patients with IHCC with a poor prognosis.

Upregulated 5-LO expression is found in several types of cancer and has been shown to be related to increased tumorigenesis [39]. A previous study revealed that the CAFs with high FAP expression promoted IHCC cell stemness via downstream 5-LO/leukotriene B4 signaling [14]. Our study demonstrated a significant association between high FAP expression and the number of 5-LO positive cells in the intratumoral region. Hence, high intratumoral expression of 5-LO was closely associated with high FAP expression and may be associated with tumor malignancy in patients with IHCC.

The present study had several limitations. First, this study was a retrospective analysis of data from a single institute with a small sample size. Second, this study only evaluated the association between histopathology and survival, without in vitro and in vivo research. Third, the cutoff values of FAP and SMA may vary between populations. In this study, the cutoff values were established based on the patients from a single hospital. Different patient populations may have different cutoff values; thus, a multicenter study or studies of other patient populations are needed to validate the cutoff values. However, the current study was limited to only patients with IHCC who had undergone curative resection; therefore, we believe that our study provides useful information for patients with IHCC.

In conclusion, high intratumoral expression of FAP was significantly associated with worse survival, suggesting that intratumoral FAP expression represents malignant progression in patients with IHCC. Therefore, intratumoral FAP expression may be useful as a prognostic factor in curatively resected IHCC, and may be an indicator of additional postoperative therapy.

### Electronic supplementary material

Below is the link to the electronic supplementary material.

Supplementary Material 1

## Data Availability

The datasets used and/or analysed during the current study available from the corresponding author on reasonable request.
