# Supplementary material for: Association between high expression of intratumoral fibroblast activation protein and survival in patients with intrahepatic cholangiocarcinoma
Source: BMC Gastroenterol. 2023 Nov 28;23:415. doi: 10.1186/s12876-023-03012-x (PMC10683315; doi:10.1186/s12876-023-03012-x)
Supplement: Supplementary file 1 — Supplementary Material 1 [file 12876_2023_3012_MOESM1_ESM.docx]

**Original article**

**Tumor central tumor FAP positive** **expression on survival in patients with intrahepatic cholangiocarcinoma**

Yuhei Waki, MD, Yuji Morine, MD, PhD, Chie Takasu, MD, PhD, Hiroki Teraoku, MD, PhD, Shinichiro Yamada, MD, PhD, Yu Saito, MD, PhD, Tetsuya Ikemoto, MD, PhD, Mitsuo Shimada, MD, PhD

Department of Digestive and Transplant Surgery, Tokushima University, Tokushima, Japan

**Correspondence:** Dr. Yuji Morine, MD, PhD

Department of Surgery, Institute of Biomedical Sciences, Tokushima University Graduate School, 3-18-15 Kuramoto-cho, Tokushima, 770-8503, Japan.

E-mail: ymorine@tokushima-u.ac.jp; Tel.: +81-088-633-7139; Fax: +81-088-633-9698


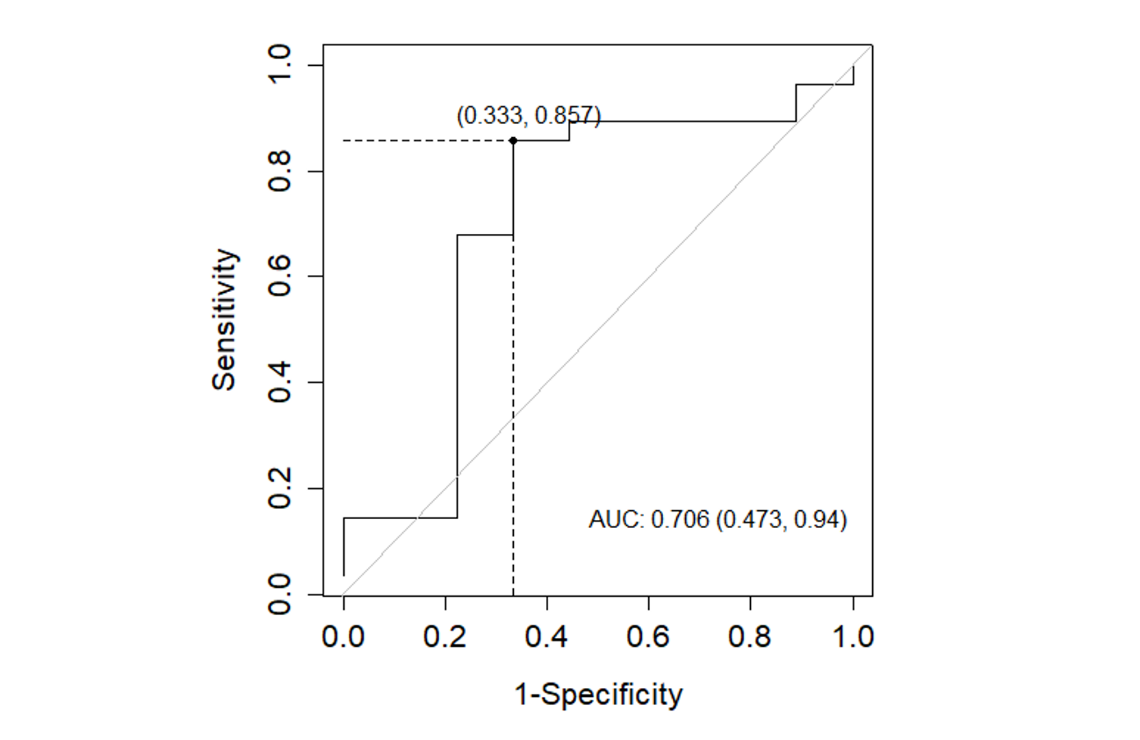


**Supplemental Figure. 1**

Receiver operating characteristic curve to identify the appropriate cut-off value of intratumoral FAP expression to predict overall survival. The area under the curve (AUC) was 0.706 and the threshold of intratumoral FAP expression was 11.8 % with sensitivity of 85.7% and specificity of 33.3%.
